# Hospitalization Risk for Medicare Beneficiaries With Nontuberculous Mycobacterial Pulmonary Disease

**DOI:** 10.1016/j.chest.2021.07.034

**Published:** 2021-07-24

**Authors:** D. Rebecca Prevots, Theodore K. Marras, Ping Wang, Kevin C. Mange, Patrick A. Flume

**Affiliations:** a Epidemiology and Population Studies Unit, Division of Intramural Research, National Institute of Allergy and Infectious Diseases, National Institutes of Health, Bethesda, MD; b Department of Respiratory Medicine, University Health Network and University of Toronto, Toronto, ON, Canada; c Insmed Incorporated, Bridgewater, NJ; d Division of Pulmonary, Critical Care, Allergy and Sleep Medicine, Medical University of South Carolina, Charleston, SC

**Keywords:** hospitalization, nontuberculous mycobacterial pulmonary disease, US Medicare, CCI, Charlson Comorbidity Index, ICD-9-CM, *International Classification of Diseases, Ninth Revision*, *Clinical Modification*, ICD-10-CM, *International Classification of Diseases*, *Tenth Revision*, *Clinical Modification*, IRR, incidence rate ratio, NTM, nontuberculous mycobacteria, NTM-PD, nontuberculous mycobacterial pulmonary disease

## Abstract

**Background:**

Nontuberculous mycobacterial pulmonary disease (NTM-PD) is an uncommon mycobacterial infection characterized by worsening lung function and increased health care resource utilization; however, the overall risk for hospitalization among patients with NTM-PD remains unclear.

**Research Question:**

What is the hospitalization risk among older adults with NTM-PD?

**Study Design and Methods:**

A retrospective, nested, case-control study was conducted by using the Medicare claims database. Cases were defined as patients with ≥ 2 NTM-PD claims ≥ 30 days apart between January 1, 2007, and December 31, 2015. The study included individuals aged ≥ 65 years with ≥ 12 months of continuous enrollment in both Parts A and B before the first NTM-PD diagnosis. Cases were matched 1:2 to Medicare beneficiaries without NTM-PD (control subjects) according to age and sex. Hospitalizations following the first NTM-PD claim were compared between case and control subjects by using univariate and multivariate analyses.

**Results:**

A total of 35,444 case subjects and 65,467 matched control subjects (mean age, 76.6 years; 70% female; ≥ 87% White) were identified. Baseline comorbidities, particularly pulmonary comorbidities, were more common in case subjects than in control subjects (81.1% vs 17.7% for COPD; 44.6% vs 0.6% for bronchiectasis). All-cause hospitalization was observed in 65.7% of case subjects and 44.9% of control subjects. Unadjusted annual hospitalization rates were significantly (*P* < .05) greater among case subjects than control subjects. Case subjects also had a significantly shorter time to hospitalization than control subjects. The increased burden due to hospitalization was reflected in multivariate analysis adjusting for baseline comorbidities. All-cause hospitalization in patients with NTM-PD relative to control subjects was 1.2 times more likely (relative risk, 1.23; 95% CI, 1.21-1.25; *P* < .0001) with a 46% greater hazard (hazard ratio, 1.46; 95% CI, 1.43-1.50; *P* < .0001).

**Interpretation:**

Patients with NTM-PD were significantly more likely to be hospitalized, had greater annualized hospitalization rates, and had shorter time to hospitalization than age- and sex-matched control subjects without NTM-PD. These findings highlight the significantly increased burden of hospitalizations among patients with NTM-PD.

## Graphical Abstract

**Figure:**
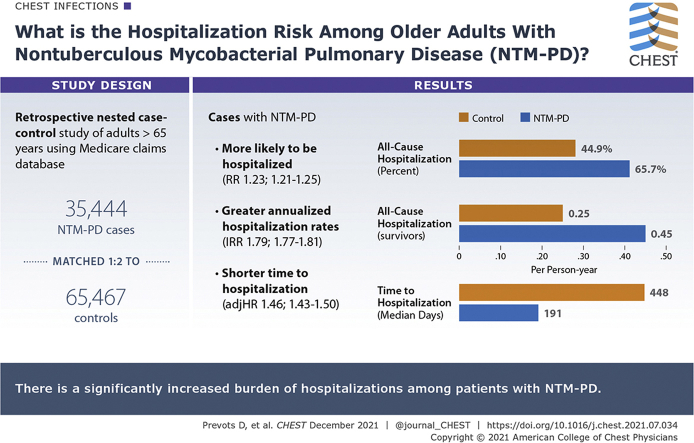

**Take-home Points**

**Study Question:** What is the hospitalization risk among older adults with NTM-PD?

**Results:** In this nested case-control study of Medicare beneficiaries (2007-2015), both univariate and multivariate analysis adjusting for baseline comorbidities show that patients with NTM-PD had an increased risk of all-cause hospitalization vs control subjects (relative risk, 1.23; 95% CI, 1.21-1.25; *P* < .0001), higher annual hospitalization rates than control subjects (IRR, 1.79; 95% CI, 1.77-1.81; *P* < .001 for all-cause hospitalization), and a significantly greater risk for sooner all-cause hospitalization relative to control subjects at any time during follow-up (hazard ratio, 1.46; 95% CI, 1.43-1.50; *P* < .0001).

**Interpretation:** Patients with NTM-PD were significantly more likely to be hospitalized, had greater annualized hospitalization rates, and had shorter time to hospitalization than age- and sex-matched control subjects without NTM-PD, highlighting the need for careful medical management of these patients and clinical management strategies that may reduce excess hospitalizations.

Nontuberculous mycobacterial pulmonary disease (NTM-PD) is a rare, chronic, progressive, and potentially debilitating condition that has become an increasingly common diagnosis.^1^, ^2^, ^3^, ^4^ In a recent analysis of a large US health insurance database, among US Medicare Advantage (Part C) patients, the annual prevalence of NTM-PD increased from 19.8 to 43.1 cases per 100,000 population from 2008 to 2015.^5^

NTM-PD is known to lead to impaired pulmonary function and quality of life and an increased risk of mortality.^1^, ^2^, ^3^, ^4^^,^^6^ In addition to the symptoms that affect daily life,^7^^,^^8^ the acute worsening of symptoms and exacerbations associated with NTM-PD can lead to hospitalization, as well as prolonged treatment, both contributing to substantial health care expenditures.^9^

In an analysis of national hospital discharge data for the United States from 1998 through 2005, the average annual prevalence of non-AIDS NTM-PD-associated hospitalizations among persons aged 70 to 79 years was 7.6 per 100,000 for men and 9.4 per 100,000 for women, with lower rates observed for younger individuals.^10^ In a study from Germany, patients with NTM-PD had five times more hospital days compared with matched control subjects over 39 months of follow-up.^11^ However, the risk of hospitalization among patients with NTM-PD remains unclear. The objective of the current study was to estimate the risk of hospitalizations associated with NTM-PD among US Medicare beneficiaries. Our null hypothesis was that the case subjects and control subjects have equal hospitalization risk, and the statistical analysis would later reject the null hypothesis.

## Subjects and Methods

### Study Population

We conducted a nested case-control study using the 100% US Medicare Parts A and B administrative claims database for January 1, 2007, through December 31, 2015. This population-based database includes beneficiaries in all 50 US states. A case of NTM-PD was defined as ≥ 2 claims with a diagnostic code for NTM-PD (*International Classification of Diseases, Ninth Revision, Clinical Modification* [ICD-9-CM] 031.0; *International Classification of Diseases, Tenth Revision, Clinical Modification* [ICD-10-CM] A31.0) that were ≥ 30 days apart.^5^^,^^12^ We also restricted our analysis to patients with NTM-PD who were aged ≥ 65 years at the time of first diagnosis of NTM-PD. Control subjects were defined as Medicare beneficiaries without any NTM-PD claims during the study period. Each case subject was matched to two control subjects according to age (±0.5 years) and sex. The index date for each NTM-PD case was defined as the date of the first NTM-PD claim; the index date for each case was assigned to the matched control subjects. Both case subjects and matched control subjects were required to have at least 12 months of continuous coverage of Medicare Parts A and B^13^ prior to the index date.

### Study Variables

Comorbid conditions for NTM-PD case subjects and matched control subjects were identified based on ICD-9-CM and ICD-10-CM codes from Medicare Parts A and B claims during the baseline period (ie, the 12 months prior to the index date). In addition, the Charlson Comorbidity Index (CCI) was calculated based on baseline diseases and health conditions to characterize patients' overall disease burden.^14^^,^^15^ A CCI score of zero indicates that there were no comorbidities, and a higher score predicts an increased risk of mortality and use of health resources.

Hospitalizations were accrued over the follow-up period, which was defined as the interval between the index date and the end of the study period (December 31, 2015), the last day prior to enrollment in Medicare Advantage (Part C), or death, whichever was earliest. Over the follow-up period, parameters related to hospitalization were estimated, including the proportion of patients with hospitalizations, the incidence rate of hospitalizations, the risk of hospitalizations, and the time to hospitalization, comparing NTM-PD case subjects vs control subjects for all-cause, COPD-related, pneumonia-related, and other pulmonary disease-related hospitalizations. Causes associated with a hospitalization were identified from the first five diagnostic codes of the inpatient claim (up to 25 diagnostic codes per inpatient claim were available in Medicare claims). When the first five diagnostic codes included more than one cause of interest, the assignment of relevant cause for that hospitalization claim followed the order of COPD, pneumonia, and other pulmonary diseases. Time to hospitalization was measured as the interval between the index date and the date of the first hospitalization during the follow-up period.

### Statistical Analysis

The study includes descriptive analysis of demographic and clinical characteristics of patients with NTM-PD and the age- and sex-matched control subjects, as well as an assessment of hospitalizations accrued over the follow-up period. We calculated the proportions of individuals with hospitalizations, the annualized hospitalization rates, and the time to hospitalizations with both univariate and multivariate analysis. Statistical significance was assessed at *P* < .05.

Categorical variables are presented as the count and percentage; continuous variables are summarized through mean ± SD. Univariate analysis included the two-sided χ^2^ test for categorical variables and the two-sided Student *t* test for continuous variables.

Poisson regression was used to estimate the unadjusted annual incidence rates of hospitalizations for NTM-PD case subjects and control subjects as well as the incidence rate ratio (IRR) of case subjects vs control subjects. Rates were estimated separately for persons who survived the follow-up period (follow-up ended due to enrolling in Medicare Advantage or data cutoff) and those who did not (follow-up ended due to death), as more hospitalizations prior to death are often observed independent of the NTM-PD status. Rates and the IRRs for all-cause, COPD-related, pneumonia-related, and other pulmonary disease-related hospitalizations are reported.

The relative risk of hospitalization was estimated by using Poisson regression, with the dependent variable defined as a Boolean variable, indicating hospitalization status. Both unadjusted and adjusted relative risks for all-cause hospitalization controlling for baseline comorbidities are reported.

Time to hospitalization was described with Kaplan-Meier survival curves stratified according to group (NTM-PD vs control). Censoring events were death, data cutoff, or enrolling in Medicare Advantage. The log-rank test was used to assess the significance of the differences in the time to hospitalization between the two groups. The hazard ratio of NTM-PD relative to control subjects was estimated by using the Cox proportional hazards model with time to all-cause hospitalization as the dependent variable, controlling for baseline clinical factors.

Statistical analyses were conducted by using SAS Enterprise Guide (version 7.15 HF3 [7.100.5.6132; 64-bit]; SAS Institute Inc).

## Results

### Patient Demographic and Clinical Characteristics

**Figure 1:**
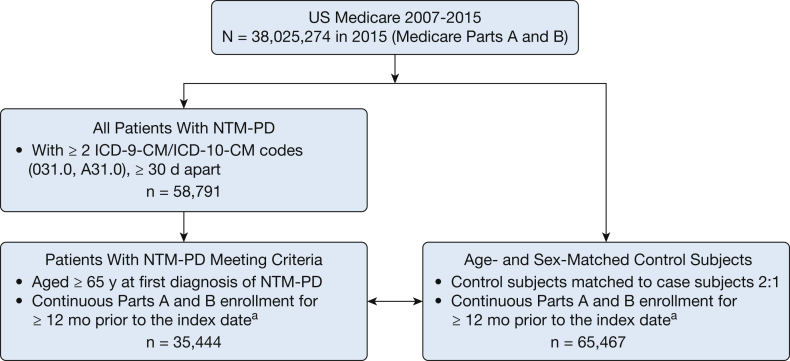
Study cohort. ^a^Index date was defined as the date the first NTM-PD diagnostic code (ICD-9-CM 031.0 or ICD-10-CM A31.0) was received for patients with NTM-PD, and this date was assigned to the matched control subjects as their index date. ICD-9-CM = *International Classification of Diseases, Ninth Revision, Clinical Modification;* ICD-10-CM = *International Classification of Diseases, Tenth Revision, Clinical Modification*; NTM-PD = nontuberculous mycobacterial pulmonary disease.

An overview of the study sample construction is provided in Figure 1. From 2007 through 2015, a total of 58,791 Medicare Parts A and B beneficiaries fulfilled the NTM-PD case definition (≥ 2 claims for NTM-PD ≥ 30 days apart); among these patients, 35,444 patients were ≥ 65 years old and had ≥ 12 months of continuous enrollment in Parts A and B prior to the index date and thus comprised the NTM-PD cohort.

The baseline characteristics of the NTM-PD cohort (n = 35,444) and the matched control subjects (n = 65,467) are provided in Table 1. The mean age was 76.6 years, and the majority were female (70.0%) and White (≥ 87%). The mean CCI score was higher in the NTM-PD cohort (2.3 ± 1.5) than in matched control subjects (1.2 ± 1.4). Most comorbidities were more common in the NTM-PD cohort than in the matched control subjects. Baseline differences were especially pronounced for pulmonary comorbidities, with proportions for the NTM-PD vs control groups being 81.1% vs 17.7% for COPD, 54.5% vs 6.6% for pneumonia, 44.6% vs 0.6% for bronchiectasis, 33.3% vs 4.5% for exacerbation of respiratory disease (acute or chronic), 27.3% vs 8.0% for asthma, and 19.4% vs 3.7% for empyema. The proportions of patients with these comorbidities were significantly different (two-sided χ^2^ test, *P* < .01).

**Table 1:** Demographic and Clinical Characteristics During the 12-Month Period Prior to the Index Date (Baseline)

Baseline Variable	NTM-PD (n = 35,444)	Control Subjects (n = 65,467)	*P* Value^a^
Age on index date, mean ± SD, y	76.6 ± 6.8	76.6 ± 6.8	
Female sex, % (n)	69.8 (24,743)	70.8 (46,320)	
Race/ethnicity, % (n)			
White	91.0 (32,269)	87.0 (56,978)	
Black	2.8 (996)	7.4 (4,861)	
Asian	3.0 (1,047)	1.8 (1,187)	
Hispanic	0.9 (328)	1.6 (1,065)	
Other, Unknown, or North American Native	2.3 (804)	2.1 (1,376)	
CCI, mean ± SD	2.3 ± 1.5	1.2 ± 1.4	<.0001
Comorbid conditions, % (n)			
COPD	81.1 (28,735)	17.7 (11,587)	<.0001
Hypertension	71.2 (25,232)	71.8 (47,004)	<.0001
Hyperlipidemia	67.5 (23,925)	66.1 (43,246)	<.0001
Pneumonia-related exacerbations	54.5 (19,299)	6.6 (4,348)	<.0001
Bronchiectasis	44.6 (15,813)	0.6 (421)	<.0001
GERD	35.6 (12,634)	19.7 (12,900)	<.0001
Acute or chronic exacerbations of respiratory disease	33.3 (11,792)	4.5 (2,962)	<.0001
Coronary artery disease	32.3 (11,430)	23.2 (15,185)	<.0001
Cancer	27.9 (9,897)	14.1 (9,208)	<.0001
Asthma	27.3 (9,691)	8.0 (5,212)	<.0001
Idiopathic interstitial lung disease	25.1 (8,903)	1.8 (1,203)	<.0001
Peripheral vascular disease	24.0 (8,498)	17.3 (11,341)	<.0001
Diabetes without complication	21.4 (7,577)	28.6 (18,726)	<.0001
Congestive heart failure	20.4 (7,243)	13.3 (8,717)	<.0001
Smoking history	20.4 (7,228)	5.4 (3,539)	<.0001
Cerebrovascular disease	19.4 (6,861)	16.1 (10,528)	<.0001
Empyema	19.4 (6,861)	3.7 (2,419)	<.0001
Renal disease	13.0 (4,592)	10.8 (7,089)	<.0001
Connective tissue disease-rheumatic disease	11.0 (3,883)	4.9 (3,187)	<.0001
TB	9.5 (3,356)	0.1 (44)	<.0001
Lung cancer	9.1 (3,210)	1.1 (692)	<.0001
Hemoptysis	8.5 (3,015)	0.2 (139)	<.0001
Myocardial infarction	8.3 (2,924)	5.0 (3,262)	<.0001
Chronic pulmonary heart disease	7.0 (2,488)	2.4 (1,557)	<.0001
Diabetes with complication	5.8 (2,044)	9.0 (5,894)	<.0001
Immune system disorder	5.6 (1,978)	1.0 (603)	<.0001
Pneumoconiosis	5.1 (1,790)	0.8 (547)	<.0001
Metastatic carcinoma	4.7 (1,654)	1.6 (1,042)	<.0001
Idiopathic interstitial pneumonia	3.9 (1,381)	0.2 (157)	<.0001
Aspergillosis and other fungal infections	3.1 (1,090)	0.1 (34)	<.0001
Dementia	2.9 (1,012)	5.3 (3,497)	<.0001
Peptic ulcer disease	2.9 (1,012)	1.7 (1,118)	<.0001
Idiopathic pulmonary fibrosis	1.3 (446)	0.1 (52)	<.0001
Organ transplant (kidney, heart, lung, or liver)	1.0 (360)	0.2 (129)	<.0001
Moderate or severe liver disease	0.7 (249)	0.2 (153)	<.0001
Hypersensitivity pneumonitis	0.7 (247)	0.1 (38)	<.0001
HIV/AIDS	0.6 (220)	0.1 (46)	<.0001
Coccidioidomycosis	0.5 (192)	0.0 (23)	<.0001
Sarcoidosis	0.5 (173)	0.0 (20)	<.0001
Cystic fibrosis	0.1 (40)	0.0 (5)	<.0001

The comorbidities included in the multivariate analyses (Poisson regression model and Cox proportional hazards model): asthma, bronchiectasis, COPD, idiopathic interstitial lung disease, TB, cancer, cerebrovascular disease, chronic pulmonary heart disease, congestive heart failure, connective tissue disease-rheumatic disease, coronary artery disease, dementia, diabetes with complication, diabetes without complication, gastroesophageal reflux disease (GERD), hyperlipidemia, hypertension, metastatic carcinoma, moderate or severe liver disease, myocardial infarction, organ transplant (kidney, heart, lung, liver), peptic ulcer disease, peripheral vascular disease, and renal disease. CCI = Charlson Comorbidity Index; NTM-PD = nontuberculous mycobacterial pulmonary disease.

a *P* values for categorical values (comorbid conditions) were from the two-sided χ^2^ test; *P* values for continuous variables (CCI) were from the two-sided Student *t* test.

The mean follow-up time was 3.1 ± 2.2 years for patients with NTM-PD and 3.3 ± 2.2 years for control subjects. Proportions of individuals with hospitalizations, annualized hospitalization rates, and time to hospitalizations accrued over the follow-up period are presented in the following sections.

### Proportions and Risk of Hospitalization

**Figure 2:**
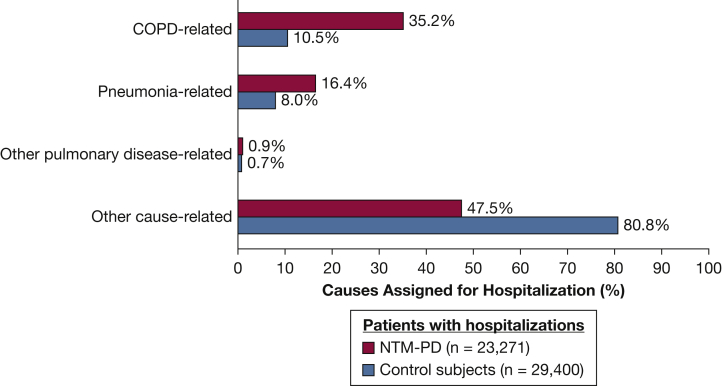
Distribution of the service provider-assigned causes for the first all-cause hospitalization in NTM-PD cases and control subjects. NTM-PD = nontuberculous mycobacterial pulmonary disease.

All-cause hospitalization was observed in 65.7% (n = 23,271) of patients with NTM-PD and 44.9% (n = 29,400) of control subjects during follow-up. Of the first all-cause hospitalizations following the index date, 52.5% were pulmonary related (35.2% COPD; 16.4% pneumonia) in the NTM-PD group, compared with 19.2% in the control group (10.5% COPD; 8.0% pneumonia) (Fig 2). The unadjusted relative risk of hospitalization associated with NTM-PD relative to control subjects was 1.98 (95% CI, 1.97-2.00; *P* < .0001), indicating that patients with NTM-PD were nearly two times more likely to have a hospitalization than the control subjects. After controlling for baseline comorbidities (Table 1), patients with NTM-PD were at a 1.2-fold increased risk of all-cause hospitalization compared with control subjects (relative risk, 1.23; 95% CI, 1.21-1.25; *P* < .0001).

### Annual Incidence Rate of Hospitalization

**Figure 3:**
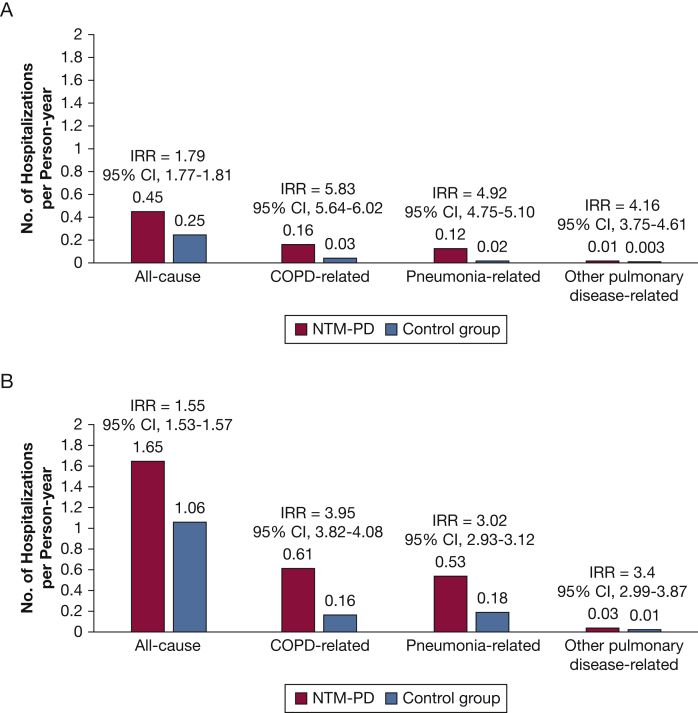
Annual incidence rate of hospitalization stratified according to survival status for participants who survived (A) or died during follow-up (B). Hospitalization rates were significantly greater in the NTM-PD group vs the control group (*P* < .001, for all comparisons). IRR = incidence rate ratio; NTM-PD = nontuberculous mycobacterial pulmonary disease.

The annual incidence rates of hospitalization were estimated separately based on the survival status at the end of the follow-up period for both the patients with NTM-PD and the control subjects. By the end of the follow-up period, 30.1% (n = 10,666) of patients with NTM-PD died, compared with 18.6% (n = 12,156) of control subjects; mean follow-up time was 2.3 ± 1.8 years for NTM-PD and 2.6 ± 1.9 years for control subjects. For those who survived at the end of follow-up, the mean follow-up time was 3.4 ± 2.2 years for patients with NTM-PD and 3.5 ± 2.2 years for control subjects. Figure 3 displays the annual incidence rate of hospitalizations (number of hospitalizations per person-year) for all-cause, COPD-related, pneumonia-related, or other pulmonary disease-related hospitalizations, among those who survived (Fig 3A) and those who died at the end of follow-up (Fig 3B). Among people who survived, the incidence rates were significantly higher among patients with NTM-PD relative to control subjects, for both all-cause and pulmonary-specific hospitalizations. For example, the incidence rate of all-cause hospitalization in patients with NTM-PD was 1.79 times the rate in control subjects (0.45 per person-year in the NTM-PD group compared with 0.25 per person-year in the control group; IRR, 1.79; 95% CI, 1.77-1.81; *P* < .001). Hospitalizations related to pulmonary conditions had a significantly higher incidence in patients with NTM-PD relative to control subjects. Similar trends of significantly higher incidence rates associated with NTM-PD were also observed among the individuals who had died at the end of follow-up. For example, the annual incidence rate for all-cause hospitalization was 1.65 in patients with NTM-PD compared with 1.06 in the control group (IRR, 1.55; 95% CI, 1.53-1.57; *P* < .001). Regardless of survival status during follow-up, differences in annual rates between the NTM-PD and control groups were approximately threefold or greater for hospitalizations related to COPD, pneumonia, or other pulmonary diseases, with the greatest difference in COPD-related hospitalization admissions.

### Time to All-Cause Hospitalization

**Figure 4:**
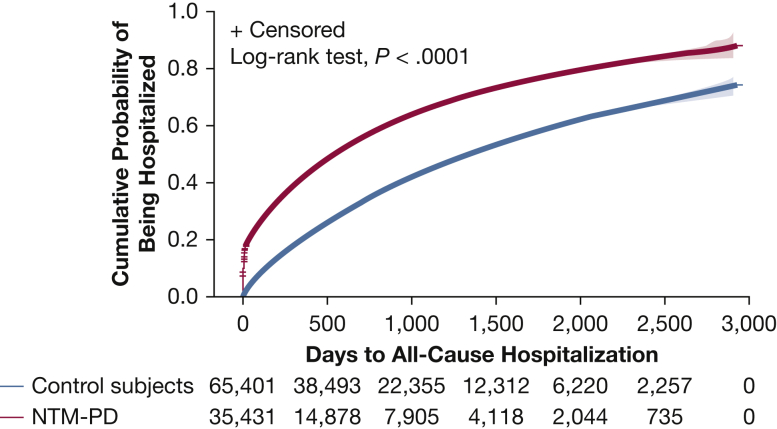
Kaplan-Meier curve of time to all-cause hospitalization. Number of subjects at risk and 95% Hall-Wellner bands are shown. NTM-PD = nontuberculous mycobacterial pulmonary disease.

Figure 4 presents the Kaplan-Meier survival curves for all-cause hospitalization, showing that the time to hospitalization was significantly shorter in patients with NTM-PD than in control subjects during the follow-up period. The median (interquartile range) time to all-cause hospitalizations was 191 (16-598) days among patients with NTM-PD vs 448 (172-924) days among control subjects. After adjusting for baseline clinical characteristics (Table 1) by using the Cox proportional hazards model, patients with NTM-PD remained at a 46% higher risk of all-cause hospitalization compared with control subjects (hazard ratio, 1.46; 95% CI, 1.43-1.50; *P* < .0001).

## Discussion

We found that among Medicare beneficiaries aged ≥ 65 years, the risk of all-cause hospitalization in patients with NTM-PD was significantly increased relative to age- and sex-matched control subjects. Overall hospitalization and pulmonary disease-related hospitalization rates were markedly higher for both patients who survived throughout the follow-up period and for those who died during the follow-up period. Pulmonary-related comorbidities were more common in patients with NTM-PD at baseline, accounted for more first all-cause hospitalizations, and likely contributed to the higher rate of all-cause hospitalization. Notably, after adjustment for confounding baseline comorbidities, compared with the control group, patients with NTM-PD had a greater than threefold increased risk of all-cause hospitalization and a 46% higher risk of having all-cause hospitalization sooner than control subjects at any time following the diagnosis of NTM-PD.

The increased risk of hospitalization attributable to NTM-PD is supported by other NTM-PD hospitalization studies as well as by studies that examined NTM-associated mortality. Hospitalizations are a marker of disease severity, as people who are hospitalized are more likely to die. One recent autopsy study showed that NTM can cause progressive, fatal disease.^16^ Similarly, in a national nested case-control study of patients treated by the Veterans Health Administration, patients had both an increased risk of outpatient visits and an increased risk of mortality, after adjusting for COPD, structural lung diseases, and immunomodulatory factors.^17^ In addition, Marras et al^18^ identified an elevated mortality risk associated with NTM in a propensity score-matched, population-based study in Ontario. These findings (NTM-related mortality) suggest a true increased risk that is attributable to NTM. This increase in risk is not necessarily a result of progression of NTM-PD. Instead, the increase in overall mortality likely stems from a contributory effect, resulting in a loss of physiological resilience and increased fragility. In addition, patients identified by using diagnostic codes as well as by microbiologic criteria have an increased risk of both hospitalization and mortality, suggesting a true association and not a systematic bias among patients identified by using diagnostic codes.

The high percentages of patients with COPD (81%) and bronchiectasis (44%) have been reported in analyses of Medicare data from an earlier period (1997-2007)^3^; although these estimates are higher than expected, they likely represent a coding issue specific to COPD. As previously noted, COPD is typically defined to include emphysema and chronic bronchitis; other conditions such as asthma and bronchiectasis, however, are also sometimes categorized as COPD by physicians and may have been coded as such in some Medicare claims analyzed here, resulting in inflated estimates of patients with NTM-PD identified through diagnostic codes.^19^

The risk of hospitalization among people with NTM-PD indicates a very high burden of disease and associated morbidity. Hospitalizations are associated with an increased cost relative to outpatient care, and an improved understanding of the risk of hospitalization attributable to NTM-PD will allow for a better estimation of associated costs.^9^^,^^11^ Although prior national population-based studies have described the features of patients hospitalized with NTM-PD in the United States,^10^ Germany,^11^^,^^20^ and South Korea,^21^ knowing the risk of hospitalization and the proportion hospitalized will allow increased generalizability of those findings. The cost of hospitalization among people with NTM-PD in the United States has been estimated to be US $709 million for 2010 alone.^9^

One strength of our analysis is that it provides population-based risk estimates among older adults in the United States. The current analysis was based on Medicare Parts A and B, which represented 69% of total Medicare enrollment in 2015; the majority (83%-84%) of Medicare Parts A and B enrollees were enrollees aged ≥ 65 years in 2010 to 2015.^13^ Our findings may not be generalizable to Medicare Part C or commercial health care plan populations, as these were excluded from our analysis. However, this sample offers real-world insights into a large nationwide population of older adults in the United States.

The primary limitation of the current study is that the ICD-9-CM/ICD-10-CM codes for NTM-PD have a low sensitivity, resulting in some underdiagnosis. The ICD-9-CM codes for NTM-PD have been evaluated in several studies, and even though their sensitivity is low,^22^^,^^23^ the positive predictive value is high among patients with microbiologically confirmed NTM-PD. Sensitivity is estimated to range from 27% to 69.9% in people with microbiologically confirmed NTM-PD,^23^ with a positive predictive value of approximately 63.2% to 82%.^23^^,^^24^ Positive predictive value is increased when using two ICD codes, as we have done in this study. Undercoding of NTM-PD in the population could lead to underestimating the total number of hospitalizations in patients with NTM-PD,^23^ but the effect on the current study is not clear. If undercoding is not associated with severity of NTM-PD or other factors that may be associated with hospitalization, then we do not think that a bias would result. If an association between undercoding and either the severity of NTM-PD or other factors associated with hospitalization were present, then a bias in either direction could be an issue. Because we lack a rationale to relate undercoding to one of these factors, we assume that it occurs at random and that the use of diagnostic codes does not bias our estimates. Despite potential limitations associated with using ICD-9-CM/ICD-10-CM diagnostic codes for case identification,^25^ the current analysis of patients with NTM-PD identified through diagnostic codes provides a robust, population-based estimate of the hospitalization burden for patients aged ≥ 65 years with NTM-PD in the United States and highlights the increased risk of hospitalization associated with NTM-PD.

## Interpretation

Patients with NTM-PD aged ≥ 65 years had statistically significantly more hospitalizations compared with age- and sex-matched control subjects without NTM-PD, highlighting the need for careful medical management of these patients and the need for clinical management strategies that may reduce excess hospitalizations for these patients.
